# Professional agency as a psychological mechanism linking AI integration to language teacher identity

**DOI:** 10.3389/fpsyg.2026.1808230

**Published:** 2026-06-23

**Authors:** Shengju Zou, Caixia Zuo, Zhiyong Yang

**Affiliations:** 1 College of Education Science, Northwest Normal University, Lanzhou, China; 2 School of Teacher Education, Qujing Normal University, Qujing, China; 3 Department of Educational Psychology, Lanzhou University of Arts and Science, Lanzhou, Gansu, China; 4 The Local Chronicles Office, Guang'an, China

**Keywords:** AI integration, AI self-efficacy, high school teachers, language teacher identity, moderated mediation, professional agency

## Abstract

**Introduction:**

The expanding use of classroom AI tools has made technology integration an increasingly identity-relevant aspect of teachers' work. Framed by psychological perspectives on professional identity, agency, and efficacy beliefs, this study examined whether AI integration in instruction is associated with language teacher identity among high school English teachers, whether professional agency represents an indirect pathway in this association, and whether the association between AI integration and professional agency differs by teachers' AI self-efficacy.

**Methods:**

A cross-sectional online survey was conducted with 420 in-service high school English teachers. Participants reported their AI integration practices, professional agency, AI self-efficacy for AI-supported teaching, and language teacher identity. Hypotheses were tested using regression-based conditional process analysis with bootstrapped confidence intervals.

**Results:**

AI integration was positively associated with language teacher identity. Professional agency was positively associated with teacher identity and the indirect association between AI integration and teacher identity through professional agency was statistically significant. Moreover, AI self-efficacy moderated the association between AI integration and professional agency, such that the positive relation was stronger among teachers with higher AI self-efficacy. Consistent with this pattern, the conditional indirect association between AI integration and teacher identity through professional agency increased across levels of AI self-efficacy.

**Discussion:**

These findings position AI integration as more than a technical practice, highlighting it as a psychologically meaningful context in which teachers enact influence and consolidate their professional self-understanding. The results suggest that supporting teachers in AI-mediated instructional environments requires not only technical guidance but also efforts to strengthen efficacy beliefs and agency-related psychological resources.

## 1 Introduction

Artificial intelligence has moved rapidly from a background infrastructure to an everyday presence in teachers' professional lives. In schools, AI is no longer only associated with automated scoring or learning analytics. The recent spread of large language models has made AI tools immediately usable for lesson planning, language input design, formative feedback, and classroom communication, particularly in language education where text and interaction are central (Kasneci et al., 2023; Kohnke et al., 2023). This shift has intensified interest in how AI can support instruction, but it has also raised a psychological question that is easy to overlook when attention is confined to pedagogical efficiency. When teachers integrate AI into their daily practice, they may also be renegotiating how they understand themselves as professionals, what they feel responsible for, and how much influence they believe they can exert on their work. In that sense, AI integration is not merely a technical adoption decision. It is a psychologically meaningful practice that can reshape professional self-understanding.

Research in AI in education has expanded quickly, mapping emerging applications and the changing landscape of instructional tools (Chen et al., 2020; Ouyang and Jiao, 2021; Salas-Pilco et al., 2022). Yet much of this literature has prioritized systems, learning outcomes, or instructional design, while teacher focused work often remains centered on readiness, attitudes, or training needs. A psychological account of teachers' adaptation to AI requires more than documenting whether teachers use AI. It requires clarifying how AI related practices connect to core aspects of the professional self. This is especially relevant for language teachers, whose work is intimately tied to interaction, interpretation, and the shaping of communicative environments. In language teaching, identity is commonly understood as a dynamic configuration that integrates personal dispositions, contextual expectations, and professional commitments (Pennington and Richards, 2016; Varghese et al., 2005). When new tools alter what counts as competent work, what is valued, and what is expected, language teachers may experience identity as something that must be actively constructed rather than passively possessed.

Teacher professional identity has long been conceptualized as multifaceted and developing across time and contexts (Beijaard et al., 2004; Akkerman and Meijer, 2011). A dialogical perspective further suggests that identity is sustained through internal and social conversations in which teachers position themselves relative to multiple voices, including institutional demands, colleagues, students, and broader cultural narratives about what teaching should be (Akkerman and Meijer, 2011). From this view, AI integration can become an identity relevant episode because it invites new comparisons and evaluations. Teachers may ask whether they remain the primary source of expertise, how their authority is expressed, and whether their professional values are preserved when AI participates in instructional decisions. Such questions are not purely educational. They involve psychological processes of meaning making, self evaluation, and the regulation of role related emotions.

To examine why AI integration may be associated with stronger or weaker teacher identity, the present study foregrounds professional agency as a potential psychological pathway. Professional agency is commonly described as the capacity to make choices, take stances, and exert influence in ways that shape work and professional identity (Eteläpelto et al., 2013). Agency is not simply a behavioral output. It reflects an experienced sense of authorship over one's practice, the ability to align actions with values, and the belief that one can actively construct workable conditions in a changing environment. In periods of reform or technological change, teachers may differ sharply in whether they experience themselves as implementing external demands or as shaping practice in ways that remain coherent with their professional self. Under this framing, AI integration may be positively related to identity when it is accompanied by a sense of agentic control and purposeful use, rather than mere compliance or substitution.

The link between technology integration and teachers' psychological functioning has been discussed in the broader literature on teacher change. Teachers' confidence, beliefs, and professional culture intersect with how technologies are taken up and how meaningful such integration becomes in practice (Ertmer and Ottenbreit-Leftwich, 2010). In parallel, school level conditions that support autonomy, competence, and relatedness can facilitate higher quality integration (Chiu, 2022). These insights suggest that AI integration should be viewed as embedded in motivational and self regulatory processes. When teachers perceive that AI supported practices allow them to pursue valued goals, respond flexibly to students, and refine their instructional judgement, integration may be experienced as an extension of professional agency. When AI use is experienced as externally imposed, confusing, or threatening to competence, integration may instead constrain agency.

A further psychological boundary condition is teachers' AI self efficacy. Self efficacy theory argues that beliefs about capability shape how people appraise task demands, sustain effort, and transform challenges into opportunities for mastery (Bandura, 1977). In teaching contexts, efficacy beliefs have been shown to relate to persistence, instructional engagement, and the ways teachers interpret difficulties in practice (Tschannen-Moran and Woolfolk Hoy, 2001). As AI becomes part of instructional work, domain specific efficacy beliefs may be particularly salient. Teachers who feel capable of selecting, prompting, evaluating, and ethically using AI tools may be more likely to approach AI integration as a space for professional choice and growth rather than as a source of dependence. Recent measurement work also reflects the field's growing effort to operationalise teachers' efficacy for AI supported teaching applications (Chou et al., 2023). These developments make it plausible that AI self efficacy shapes not only whether teachers use AI, but also whether AI integration can be translated into an agentic experience that reinforces professional identity.

Overall, these strands of evidence motivate a psychologically grounded model in which AI integration is related to language teacher identity directly and indirectly through professional agency, with the indirect pathway contingent on AI self efficacy. In our framework, AI integration represents teachers' high quality incorporation of AI tools into student centered instructional practices. Professional agency captures the experienced capacity to influence and intentionally shape classroom work. Language teacher identity is conceptualized as a relatively consolidated sense of oneself as a competent and committed language professional. AI self efficacy reflects confidence in enacting AI supported teaching effectively.

By testing this model among in service high school English teachers, the study contributes to the psychological literature in three ways. First, it shifts attention from AI as a classroom instrument to AI as a psychologically consequential context in which the professional self is negotiated. Second, it specifies professional agency as a mechanism linking a concrete behavioral domain, AI integration, with a central self construct, teacher identity. Third, it identifies AI self efficacy as a boundary condition that may help explain why similar levels of AI integration do not produce uniform psychological consequences across teachers. In doing so, the study aims to clarify how teachers' beliefs and agentic experiences shape identity related adaptation in an era of accelerating AI change.

## 2 Literature review and hypotheses development

### 2.1 Conceptual foundations

Artificial intelligence has rapidly expanded from back-end analytics to front-line pedagogical tools that can generate texts, provide feedback, support assessment, and personalize learning activities. Contemporary reviews suggest that the educational value of AI depends less on the mere presence of tools and more on how teachers integrate them into pedagogical reasoning, classroom interaction, and professional judgment (Chen et al., 2020; Ouyang and Jiao, 2021; Kasneci et al., 2023). In language education, early discussions of generative AI emphasize both opportunities for scaffolding language practice and the practical tensions it creates for classroom authority, assessment norms, and teachers' roles (Kohnke et al., 2023).

In the broader technology integration literature, integration quality is often conceptualized as the alignment between technology affordances and instructional goals, together with teachers' capacity to orchestrate learning processes rather than simply adopt tools (Mishra and Koehler, 2006; Ertmer and Ottenbreit-Leftwich, 2010). This perspective is consistent with recent efforts to operationalise technology integration quality as a multi-dimensional practice construct that captures pedagogical intention and classroom enactment (Siyam et al., 2025). At the same time, teachers' beliefs, confidence, and perceived capability shape whether new tools become meaningful resources for teaching or remain peripheral additions (Ertmer and Ottenbreit-Leftwich, 2010; Chiu, 2022).

Teacher professional identity, particularly in language teaching, is commonly understood as a dynamic sense of who one is as a teacher, formed through ongoing interpretation of practice, relationships, and institutional expectations (Beijaard et al., 2004; Akkerman and Meijer, 2011; Varghese et al., 2005; Beauchamp and Thomas, 2009). Such identity is not only an internal self-definition but also a socially situated and practice-based accomplishment, continually negotiated in response to reforms and new pedagogical demands (Akkerman and Meijer, 2011; Varghese et al., 2005). Because AI-supported teaching can reshape what counts as competent instruction and how professional expertise is displayed, AI integration may become a salient context in which teacher identity is strengthened, revised, or contested (Kohnke et al., 2023; Bergdahl and Sjöberg, 2025).

Professional agency provides a complementary lens for understanding teachers' responses to change. Professional agency refers to teachers' intentional capacity to influence their work and learning, including making choices, taking initiative, and transforming practice in interaction with social and organizational conditions (Eteläpelto et al., 2013; Pyhältö et al., 2015; Pantić, 2015). Agency is therefore closely tied to teachers' learning, adaptability, and sense of ownership over pedagogical decisions (Pietarinen et al., 2016; Biesta et al., 2015).

Building on these perspectives, the present study proposes that AI integration is associated with language teacher identity both directly and indirectly through professional agency, and that AI self-efficacy strengthens the pathway from AI integration to professional agency.

### 2.2 AI integration and language teacher identity

Teachers' identities develop through the meanings they attach to their work and the kinds of practices they are able to enact over time (Beijaard et al., 2004; Akkerman and Meijer, 2011). When teachers incorporate new tools into instruction, they often need to reinterpret what constitutes good teaching, how expertise is demonstrated, and how professional responsibilities are distributed between the teacher and technology (Kasneci et al., 2023; Kohnke et al., 2023). In language classrooms, where communication, feedback, and assessment are central, AI-supported tools can alter interaction patterns and teachers' perceived professional value, which may in turn shape teachers' sense of professional self-understanding (Varghese et al., 2005; Bergdahl and Sjöberg, 2025).

High-quality integration is typically associated with purposeful use that supports learning goals and positions the teacher as an orchestrator of learning processes (Mishra and Koehler, 2006; Ertmer and Ottenbreit-Leftwich, 2010). Such enactment can strengthen teachers' professional identity by reinforcing perceptions of competence, relevance, and pedagogical leadership in a changing instructional environment (Chiu, 2022; Siyam et al., 2025). Empirical work on teachers' engagement with AI in schools further suggests that teachers who perceive AI as compatible with their pedagogical aims and who experience confidence in using AI report more positive professional meanings attached to AI-supported teaching (Bergdahl and Sjöberg, 2025). Overall, integrating AI into instruction is likely to be associated with a stronger and more consolidated sense of language teacher identity.

*H1: AI integration in instruction is positively associated with language teacher identity*.

### 2.3 AI integration and professional agency

Professional agency is enacted when teachers initiate, adapt, and transform practice in response to emerging demands, while negotiating contextual constraints and resources (Eteläpelto et al., 2013; Pietarinen et al., 2016). AI integration is not a purely technical act. It requires teachers to make pedagogical judgments about task design, feedback standards, assessment validity, and appropriate classroom roles for AI tools (Chen et al., 2020; Ouyang and Jiao, 2021). These judgments are inherently agentic because teachers must decide how to appropriate or resist tool affordances, and how to align AI outputs with instructional values and student needs (Biesta et al., 2015; Pantić, 2015).

Research on technology-related change suggests that teachers are more likely to engage in meaningful integration when they perceive that the environment supports autonomy, competence, and professional growth, which can amplify agentic engagement with new practices (Chiu, 2022; Ertmer and Ottenbreit-Leftwich, 2010). In addition, emerging evidence indicates that teachers who actively use AI in teaching tend to report a more proactive stance toward experimenting with AI-supported practices and refining their instructional strategies (Bergdahl and Sjöberg, 2025). Therefore, teachers who integrate AI more extensively are expected to report higher professional agency.

*H2: AI integration in instruction is positively associated with teachers' professional agency*.

### 2.4 Professional agency and language teacher identity

Teacher identity is shaped through practice-based self-interpretation and through teachers' perceived ability to act meaningfully within their professional contexts (Beijaard et al., 2004; Akkerman and Meijer, 2011; Beauchamp and Thomas, 2009). Professional agency is central in this process because agency reflects teachers' sense of ownership, influence, and intentionality in their work. When teachers experience themselves as capable of making choices and shaping classroom practice, they are more likely to develop coherent narratives of professional competence and commitment (Eteläpelto et al., 2013; Pyhältö et al., 2015). In language teaching specifically, identity is often negotiated through pedagogical decisions and role positioning, making agency particularly relevant to how teachers understand themselves as legitimate and effective professionals (Varghese et al., 2005).

Agency-oriented learning in teacher communities has also been linked to reflective practice, transformation of routines, and sustained engagement with professional development, each of which supports a more consolidated professional identity (Pyhältö et al., 2015; Pietarinen et al., 2016). Accordingly, higher professional agency should be associated with stronger language teacher identity.

*H3: Teachers' professional agency is positively associated with language teacher identity*.

### 2.5 Professional agency as a mediator between AI integration and language teacher identity

The preceding arguments suggest that AI integration may influence teacher identity partly because it invites or necessitates agentic work. Integrating AI into instruction can create occasions for teachers to redesign pedagogy, evaluate tool outputs, and negotiate classroom norms around authorship and feedback (Kohnke et al., 2023; Kasneci et al., 2023). When teachers respond to these demands through intentional experimentation and reflective adjustment, they enact professional agency (Eteläpelto et al., 2013; Pietarinen et al., 2016). Through this agentic process, teachers may come to view themselves as adaptive professionals who can lead learning under new conditions, thereby strengthening professional identity (Beijaard et al., 2004; Akkerman and Meijer, 2011; Pyhältö et al., 2015).

This logic implies an indirect pathway in which AI integration contributes to identity by fostering professional agency, while allowing for the possibility that AI integration also relates to identity directly through other routes, such as perceived competence or perceived relevance of one's work (Siyam et al., 2025; Bergdahl and Sjöberg, 2025).

*H4: Professional agency mediates the association between AI integration and language teacher identity*.

### 2.6 AI self-efficacy as a moderator of the AI integration to professional agency link

Self-efficacy theory proposes that perceived capability influences whether individuals initiate behavior, persist in difficulty, and translate intentions into effective action (Bandura, 1977). In teaching, efficacy beliefs are consistently associated with greater instructional initiative and resilience, particularly in contexts that require adaptation and innovation (Tschannen-Moran and Woolfolk Hoy, 2001). Technology-related change studies similarly indicate that confidence in using tools supports teachers' willingness to experiment and to engage in deeper integration rather than superficial adoption (Ertmer and Ottenbreit-Leftwich, 2010).

AI self-efficacy extends this logic to AI-supported teaching, reflecting teachers' confidence in selecting, using, and evaluating AI tools for pedagogical purposes (Chou et al., 2024; Bergdahl and Sjöberg, 2025). When AI self-efficacy is high, teachers may be more likely to interpret AI integration as an opportunity for purposeful pedagogical action, thereby translating integration efforts into stronger professional agency. When AI self-efficacy is low, integration may remain constrained by uncertainty and reliance on limited routines, weakening the agency-related benefits of AI use. Thus, AI self-efficacy should strengthen the positive association between AI integration and professional agency.

*H5: AI self-efficacy moderates the association between AI integration and professional agency, such that the association is stronger at higher levels of AI self-efficacy*.

### 2.7 Moderated mediation

If AI self-efficacy amplifies the effect of AI integration on professional agency, and professional agency contributes to teacher identity, then the indirect association between AI integration and identity via professional agency should also depend on AI self-efficacy. In other words, the mediation pathway becomes conditional, producing a moderated mediation pattern (Hayes, 2015). In applied behavioral research, conditional indirect effects provide a useful framework for testing whether a theorized mechanism operates more strongly for individuals with greater perceived capability (Preacher and Hayes, 2008; Hayes, 2015). Therefore, we expect that the indirect effect of AI integration on language teacher identity through professional agency will be stronger among teachers with higher AI self-efficacy.

*H6: AI self-efficacy moderates the indirect association between AI integration and language teacher identity via professional agency, such that the indirect effect is stronger at higher levels of AI self-efficacy*.

**Figure 1:**
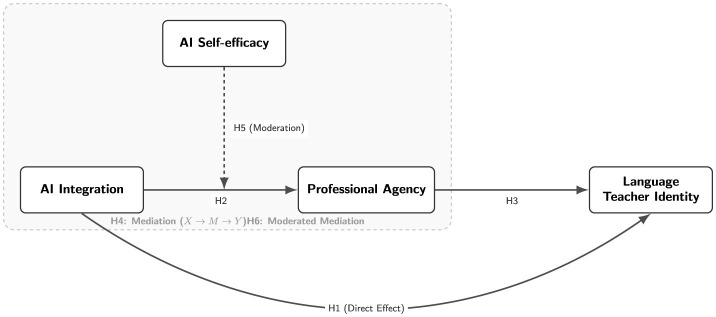
The hypothesized theoretical framework of the study.

The full conceptual model is presented in Figure 1.

## 3 Methods

### 3.1 Design and participants

We conducted a cross-sectional questionnaire study with in-service high school English teachers. A total of 493 questionnaire responses were initially submitted. During data screening, 26 incomplete responses were excluded using listwise deletion, and 47 responses were excluded because they did not meet the response-quality criteria. The final analytic sample consisted of 420 teachers, corresponding to a valid-case proportion of 85.19% among initially submitted questionnaires. After this screening process, the analytic dataset contained no item-level missing values for the focal variables and covariates. Participation was voluntary and anonymous, and informed consent was obtained before the survey began.

### 3.2 Procedure

The questionnaire was administered through Wenjuanxing, and the survey link was distributed to eligible teachers through online teacher communities and school-based English teaching groups. Eligible participants were invited to participate if they were currently working as high school English teachers. The survey included measures of AI integration in instruction, professional agency, AI self-efficacy for AI-supported teaching, and teacher professional identity, along with demographic items. When the survey language differed from the original scale language, items were translated and back-translated using standard procedures to preserve semantic equivalence (Brislin, 1970). To reduce common method bias, several procedural remedies were used. The survey was voluntary and anonymous, and participants were informed that there were no right or wrong answers. The questionnaire also psychologically separated the predictor, mediator, moderator, and outcome sections to reduce respondents' tendency to infer the study hypotheses. In addition, item stems were varied where appropriate to reduce repetitive response patterns and evaluation apprehension (Podsakoff et al., 2003).

### 3.3 Measures

All study variables were measured using 5-point Likert-type scales. Unless otherwise noted, responses ranged from 1 (strongly disagree) to 5 (strongly agree). For all multi-item measures, scale scores were computed as the arithmetic mean of item responses, consistent with common practice when using regression-based conditional process models (Hayes, 2017). The scoring approach followed the theoretically intended structure of each instrument. Specifically, the focal constructs were represented by overall mean scores because the present model tested construct-level associations among AI integration, AI self-efficacy, professional agency, and teacher professional identity. For adapted or contextually modified measures, corrected item-total correlations were inspected before scale scores were computed to ensure that each item was aligned with its corresponding scale.

AI integration was operationalized as high-quality instructional integration practices adapted to the context of AI-based tools. We drew on the high-quality technology integration practices items reported by Chiu (2022) and replaced references to technology with AI-based instructional tools while retaining the student-centered intent. The eight items captured practices such as using AI tools to provide formative assessment, to support student goal setting and self-assessment, to diagnose learning needs, and to foster collaborative learning. Because the items were adapted from a general technology integration measure to the AI-supported instructional context, we further examined the psychometric performance of the adapted scale in the present sample by conducting confirmatory factor analysis and inspecting corrected item-total correlations.

#### 3.3.1 Professional agency

Professional agency was measured with the Teachers' Professional Agency in the Classroom measure, which captures teachers' agentic engagement with their work context and classroom practice. Following prior validation work, the scale assesses agency through dimensions reflecting collaborative environment, transformative practice, and reflection in the classroom (Heikonen et al., 2017; Pyhältö et al., 2015; Soini et al., 2016). Items were rated on a 5-point scale from 1 (strongly disagree) to 5 (strongly agree), and a mean score was computed, with higher values indicating stronger perceived professional agency.

#### 3.3.2 AI self-efficacy

AI self-efficacy was measured using the AI-supported Teaching Applications Self-efficacy scale developed for high school teachers by Chou et al. (2024). The instrument conceptualizes teachers' confidence for AI-supported teaching across five facets, and items were rated on a 5-point response format. Consistent with the instrument's intended use, we computed an overall mean score, with higher values reflecting greater confidence in engaging AI-supported teaching applications.

#### 3.3.3 Teacher professional identity

Teacher identity was operationalized as teacher professional identity, conceptualized as teachers' relatively stable self-understanding of their professional role, competence, and commitment in instructional practice. We measured this construct using the Teacher Professional Identity Scale developed and validated by Zeng and Liu (2024). The scale comprises 13 items across three domains: professional self-efficacy, professional knowledge, and professional commitment. All items were rated on a 5-point Likert scale ranging from 1 (strongly disagree) to 5 (strongly agree). A mean score was computed, with higher scores indicating a more consolidated teacher professional identity. To enhance contextual fit, item wording was minimally adapted to the senior high school English-teaching context without altering the substantive meaning of the original items.

#### 3.3.4 Control variables

We included five demographic variables as covariates to reduce confounding: gender, age, highest degree, years of teaching experience, and prior AI-related training or professional development experience. Age was reported descriptively in Table 1 but was not included as a covariate in subsequent regression models because it overlapped substantially with years of teaching and did not add conceptual clarity to the analyses.

**Table 1:** Sample characteristics.

Variable	Category	*n*	Percent
Gender	Female Male	318 102	75.7 24.3
Age	20–29	86	20.5
	30–39	172	41.0
	40–49	118	28.1
	50 and above	44	10.5
Years of teaching	0–3	74	17.6
	4–9	98	23.3
	10–19	156	37.1
	20 and above	92	21.9
Highest degree	Bachelor	328	78.1
	Master	89	21.2
	Doctorate	3	0.7
School location	Urban	205	48.8
	County or town	135	32.1
	Rural	80	19.0
AI related training^a^	None	164	39.0
	Once	148	35.2
	Two or more times	108	25.7

^a^AI related training refers to any professional development focused on using AI for teaching during the past 12 months.

### 3.4 Data analysis

Analyses were conducted using IBM SPSS Statistics (IBM Corp., Armonk, NY, United States). We first computed descriptive statistics, internal consistency estimates, and zero-order correlations. Hypotheses were tested using Hayes' regression-based conditional process framework (Hayes, 2017). Our conceptual model specified AI integration as the predictor (X), professional agency as the mediator (M), teacher professional identity as the outcome (Y), and AI self-efficacy as a moderator (W) of the X to M path. This corresponds to PROCESS Model 7 (Hayes, 2017). Continuous predictors were mean-centered prior to forming interaction terms. Indirect effects and conditional indirect effects were evaluated using bootstrap confidence intervals based on 5,000 resamples, with 95 percent confidence intervals used to determine statistical significance.

To assess the risk of common method bias, we complemented the procedural remedies with a *post hoc* diagnostic, conducting Harman's single-factor test as a coarse check, interpreted alongside the procedural safeguards and the overall pattern of results (Podsakoff et al., 2003).

## 4 Results

### 4.1 Sample characteristics

Table 1 presents the demographic profile of the 420 high school English teachers included in the final analyses. The sample was predominantly female (75.7%), with most participants aged between 30 and 39 years (41.0%), followed by those aged 40–49 years (28.1%). In terms of teaching experience, the largest group reported 10–19 years of service (37.1%), whereas 17.6% had taught for 0 to 3 years and 21.9% reported 20 years or more. Most teachers held a bachelor's degree (78.1%), and nearly half worked in urban schools (48.8%). With respect to professional development related to AI, 39.0% reported no AI-related training in the past 12 months, 35.2% reported attending one training session, and 25.7% reported attending two or more sessions.

### 4.2 Descriptive statistics reliability and correlations

Table 2 shows the descriptive statistics and internal consistency estimates for the core constructs. All scales demonstrated good internal consistency, with Cronbach α values ranging from 0.884 to 0.923. On average, teachers reported moderate levels of AI integration (*M* = 3.421, SD = 0.765) and AI self-efficacy (*M* = 3.562, SD = 0.684), alongside relatively high professional agency (*M* = 3.845, SD = 0.652) and language teacher identity (*M* = 4.124, SD = 0.583).

**Table 2:** Descriptive statistics and reliability.

Construct	Items	Cronbach α	Mean	SD	Observed range
AI integration	8	0.884	3.421	0.765	1.250–5.000
AI self efficacy	15	0.923	3.562	0.684	1.000–5.000
Professional agency	10	0.891	3.845	0.652	1.800–5.000
Language teacher identity	13	0.915	4.124	0.583	2.150–5.000

All constructs were scored as item means. Higher values indicate higher levels of each construct.

In addition to Cronbach's alpha, corrected item-total correlations were inspected for all focal scales. The corrected item-total correlations were acceptable for all constructs: AI integration, 0.552–0.721; AI self-efficacy, 0.546–0.782; professional agency, 0.519–0.735; and teacher identity, 0.561–0.767. These results supported the use of overall mean scores for the four focal constructs in the subsequent regression-based conditional process analyses.

Given that the AI integration scale was adapted from a general technology integration measure, we conducted additional psychometric checks for the adapted 8-item scale. A one-factor confirmatory factor analysis was estimated to examine whether the adapted items supported the assumed unidimensional structure in the present AI-supported teaching context. The model showed good fit to the data, χ^2^(20) = 43.60, χ^2^/df = 2.18, CFI = 0.971, TLI = 0.962, RMSEA = 0.053, and SRMR = 0.038. Standardized factor loadings ranged from 0.641 to 0.814, and corrected item-total correlations ranged from 0.552 to 0.721. As shown in Table 3, these results provide additional support for using the adapted AI integration items as a unidimensional mean score in the present analysis.

**Table 3:** Psychometric evidence for the adapted AI integration scale.

Item	Standardized factor loading	Corrected item-total correlation
AI1	0.758	0.643
AI2	0.814	0.721
AI3	0.672	0.568
AI4	0.739	0.615
AI5	0.641	0.552
AI6	0.783	0.694
AI7	0.726	0.607
AI8	0.695	0.589

Standardized factor loadings were obtained from a one-factor confirmatory factor analysis (CFA) of the adapted AI integration scale. Global model fit indices for the CFA were: χ^2^(20) = 43.60, χ^2^/df = 2.18, CFI = 0.971, TLI = 0.962, RMSEA = 0.053, SRMR = 0.038. Corrected item-total correlations were computed by correlating each item with the sum of the remaining items.

Zero-order correlations are presented in Table 4. AI integration was positively associated with AI self-efficacy (*r* = 0.582, *p* < 0.001), professional agency (*r* = 0.425, *p* < 0.001), and teacher identity (*r* = 0.342, *p* < 0.001). AI self-efficacy was also positively related to professional agency (*r* = 0.468, *p* < 0.001) and teacher identity (*r* = 0.395, *p* < 0.001). Professional agency showed the strongest bivariate association with teacher identity (*r* = 0.556, *p* < 0.001), providing preliminary support for the proposed mediation pathway.

**Table 4:** Correlations among study variables.

Variable	1	2	3	4	5	6	7	8	9
1 AI integration	(0.884)								
2 AI self efficacy	0.582^***^	(0.923)							
3 Professional agency	0.425^***^	0.468^***^	(0.891)						
4 Teacher identity	0.342^***^	0.395^***^	0.556^***^	(0.915)					
5 Gender^a^	−0.042	0.081	−0.023	−0.065	1.000				
6 Years of teaching	−0.145^**^	−0.112^*^	0.135^**^	0.264^***^	-0.052	1.000			
7 Highest degree^b^	0.064	0.035	0.121^*^	0.054	-0.021	−0.152^**^	1.000		
8 School location^c^	0.215^***^	0.142^**^	0.075	0.042	0.015	−0.041	0.092	1.000	
9 AI related training^d^	0.412^***^	0.365^***^	0.192^***^	0.125^*^	0.054	−0.065	0.042	0.154^**^	1.000

Reliabilities are shown on the diagonal in parentheses. Significance levels: ^*^p < 0.050, ^**^p < 0.010, ^***^p < 0.001.

^*a*^Gender coded as 0 = female, 1 = male.

^*b*^Highest degree coded as 1 = bachelor, 2 = master, 3 = doctorate.

^*c*^School location coded as 1 = rural, 2 = county/town, 3 = urban.

^*d*^AI related training coded as 0 = none, 1 = once, 2 = two or more times.

Among the covariates, years of teaching was negatively related to AI integration (*r* = −0.145, *p* < 0.010) and AI self-efficacy (*r* = −0.112, *p* < 0.050), but positively related to professional agency (*r* = 0.135, *p* < 0.010) and teacher identity (*r* = 0.264, *p* < 0.001). Teachers working in more urban settings reported higher AI integration (*r* = 0.215, *p* < 0.001) and higher AI self-efficacy (*r* = 0.142, *p* < 0.010). AI-related training was positively associated with AI integration (*r* = 0.412, *p* < 0.001), AI self-efficacy (*r* = 0.365, *p* < 0.001), professional agency (*r* = 0.192, *p* < 0.001), and teacher identity (*r* = 0.125, *p* < 0.050). Gender showed no significant correlations with the focal constructs.

### 4.3 Common method bias diagnostic

Because the focal variables were assessed using self-report measures, we examined whether a single latent factor appeared to account for most of the covariance among items. We conducted Harman's single-factor test by entering all 46 measurement items into an unrotated exploratory factor analysis. As shown in Table 5, eight factors with eigenvalues greater than 1 emerged, and the first factor accounted for 34.125% of the total variance. This pattern suggests that the data were not dominated by a single general factor. Because Harman's single-factor test is only a coarse diagnostic, we also inspected the zero-order correlation matrix for implausibly high intercorrelations among the focal constructs. The correlations among AI integration, AI self-efficacy, professional agency, and teacher identity ranged from 0.342 to 0.582, with the highest correlation observed between AI integration and AI self-efficacy. None of the focal construct correlations approached levels that would suggest redundancy or a single dominant response tendency. Therefore, although common method variance cannot be fully ruled out in a self-report cross-sectional survey, the combined evidence from procedural safeguards, Harman's test, and the correlation matrix inspection suggests that the main findings were unlikely to be driven solely by common method variance.

**Table 5:** Harman single factor test summary.

Indicator	Value	Criterion or reference
Total items entered	46	Not applicable
Number of factors with eigenvalues above 1	8	Not applicable
Variance explained by the first factor percent	34.125	Below 40 as a common heuristic

### 4.4 Hypothesis testing of the moderated mediation model

We tested the hypothesized first-stage moderated mediation model using PROCESS, with AI integration as the predictor, professional agency as the mediator, teacher identity as the outcome, and AI self-efficacy as a moderator of the path from AI integration to professional agency. Table 6 reports the regression results predicting professional agency. AI integration was positively associated with professional agency (*b* = 0.165, *p* = 0.003), supporting H2. AI self-efficacy also showed a positive main association with professional agency (*b* = 0.184, *p* = 0.002). The interaction between AI integration and AI self-efficacy was significant (*b* = 0.098, *p* = 0.035), indicating that the association between AI integration and professional agency depended on teachers' AI self-efficacy, consistent with H5. To evaluate the size of the moderation effect, we compared the mediator model with and without the interaction term. The interaction term explained an additional Δ*R*^2^ = 0.008 of the variance in professional agency beyond the main effects and covariates, with *F*_change(1, 411)_ = 4.47, *p* = 0.035. The full mediator model accounted for 26.8% of the variance in professional agency (*R*^2^ = 0.268).

**Table 6:** Regression results predicting professional agency.

Predictor	*b*	SE	*t*	*p*-Value	LLCI	ULCI
Constant	3.242	0.158	20.519	< 0.001	2.931	3.553
AI integration	0.165	0.054	3.055	0.003	0.058	0.272
AI self efficacy	0.184	0.058	3.172	0.002	0.069	0.299
AI integration × AI self efficacy	0.098	0.046	2.115	0.035	0.007	0.189
Gender	−0.015	0.065	−0.231	0.817	−0.143	0.113
Years of teaching	0.055	0.022	2.500	0.013	0.012	0.098
Highest degree	0.115	0.054	2.130	0.034	0.009	0.221
School location	0.025	0.036	0.694	0.488	−0.046	0.096
AI related training	0.032	0.039	0.821	0.412	−0.045	0.109
Model *R*^2^	0.268					
Model *F*_(8, 411)_	18.750, *p* < 0.001					

Professional agency is the dependent variable. AI integration and AI self efficacy were mean centered prior to computing the interaction term.

**Figure 2:**
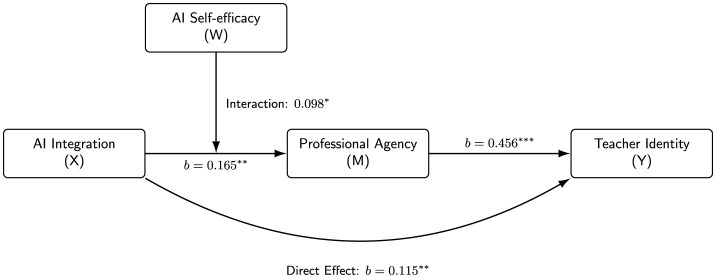
The moderated mediation model results. Covariates controlled: Gender, Years of teaching, Highest degree, School location, AI related training. Unstandardized coefficients (*b*) are reported. ^*^p < 0.05, ^**^p < 0.01, ^***^p < 0.001.

Table 7 reports the regression model predicting teacher identity. Professional agency was strongly and positively associated with teacher identity (*b* = 0.456, *p* < 0.001), supporting H3. AI integration also showed a significant direct association with teacher identity after accounting for professional agency (*b* = 0.115, *p* = 0.003). We did not use the significance of this direct association to classify the mediation pattern as partial or full. Instead, this direct association was used together with the conditional indirect associations to quantify the proportion of the model-implied total association represented by the professional-agency pathway. The outcome model explained 34.2% of the variance in teacher identity (*R*^2^ = 0.342). Figure 2 summarizes the estimated paths in the moderated mediation model.

**Table 7:** Regression results predicting teacher identity.

Predictor	*b*	SE	*t*	*p*-Value	LLCI	ULCI
Constant	3.015	0.142	21.232	< 0.001	2.736	3.294
AI integration	0.115	0.038	3.026	0.003	0.040	0.190
Professional agency	0.456	0.041	11.122	< 0.001	0.375	0.537
Gender	−0.024	0.058	−0.414	0.679	−0.138	0.090
Years of teaching	0.112	0.018	6.222	< 0.001	0.077	0.147
Highest degree	0.015	0.048	0.312	0.755	−0.079	0.109
School location	0.012	0.032	0.375	0.708	−0.051	0.075
AI related training	0.021	0.035	0.600	0.549	−0.048	0.090
Model *R*^2^	0.342					
Model *F*_(7, 412)_	30.610, *p* < 0.001					

Teacher identity is the dependent variable. AI integration is included to estimate the direct effect while accounting for the mediator.

### 4.5 Simple slopes of the interaction effect

**Figure 3:**
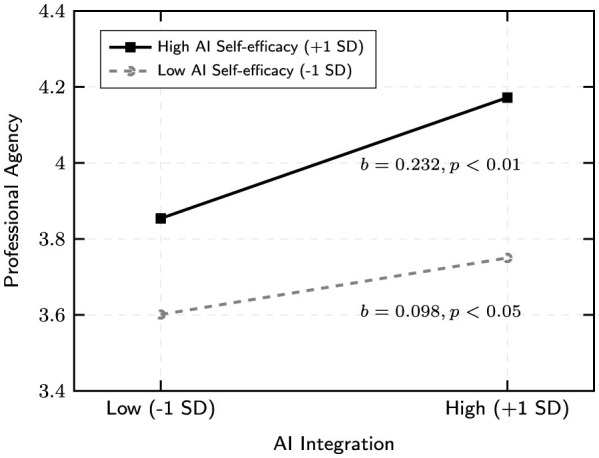
Simple slope analysis of the interaction between AI integration and AI self-efficacy on professional agency.

To clarify the nature of the interaction between AI integration and AI self-efficacy, we probed the conditional effect of AI integration on professional agency at representative levels of AI self-efficacy. As shown in Figure 3, AI integration was positively related to professional agency when AI self-efficacy was low (*b* = 0.098, *p* < 0.050). This positive association was substantially stronger when AI self-efficacy was high (*b* = 0.232, *p* < 0.010). Overall, the simple slope pattern indicates that teachers with stronger confidence in their AI-related capabilities were more likely to translate greater AI integration into higher professional agency.

### 4.6 Indirect effects conditional indirect effects and moderated mediation

**Figure 4:**
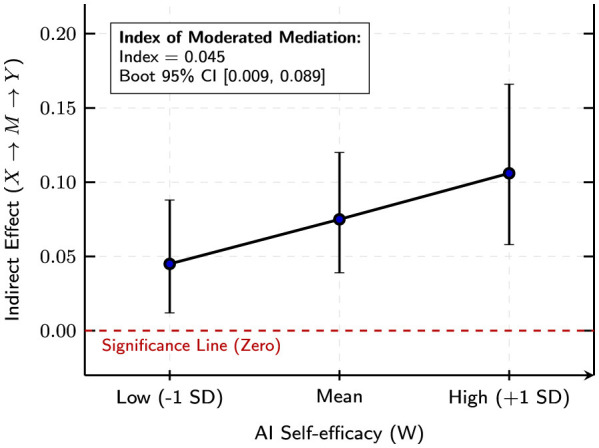
Conditional indirect effects of AI integration on teacher identity via professional agency across different levels of AI self-efficacy.

We further examined whether AI integration was linked to teacher identity indirectly through professional agency, and whether this indirect association varied as a function of AI self-efficacy. Table 8 reports the bootstrap estimates of the conditional indirect associations at low, mean, and high levels of AI self-efficacy. The indirect association was significant when AI self-efficacy was low [Indirect association = 0.045, Boot 95% CI (0.012, 0.088)], and it remained significant at the mean level of AI self-efficacy [Indirect association = 0.075, Boot 95% CI (0.039, 0.120)]. The indirect association was strongest when AI self-efficacy was high [Indirect association = 0.106, Boot 95% CI (0.058, 0.166)]. In unstandardized scale-score terms, a one-point higher AI integration score was indirectly associated with a 0.045-point higher teacher identity score through professional agency when AI self-efficacy was low, compared with a 0.106-point higher teacher identity score when AI self-efficacy was high. Thus, although the conditional indirect associations were modest in absolute size on a 5-point scale, the high-AI-self-efficacy indirect association was more than twice the low-AI-self-efficacy indirect association. This pattern suggests that AI self-efficacy meaningfully differentiated the strength of the professional-agency pathway, even though the absolute scale-score changes should be interpreted cautiously. This pattern is also illustrated in Figure 4, where the conditional indirect association increases across levels of AI self-efficacy.

**Table 8:** Conditional indirect associations and moderated mediation.

Condition	Indirect association	Boot SE	Boot LLCI	Boot ULCI	Proportion of total association (%)
AI self efficacy low (−1 SD)	0.045	0.019	0.012	0.088	28.1
AI self efficacy mean	0.075	0.021	0.039	0.120	39.5
AI self efficacy high (+1 SD)	0.106	0.028	0.058	0.166	48.0
Index of moderated mediation	0.045	0.020	0.009	0.089	–

Bootstrap confidence intervals are based on 5,000 resamples. An indirect association is supported when the bootstrap confidence interval does not include zero. The proportion of total association was computed as the conditional indirect association divided by the sum of the direct association and the conditional indirect association. The direct association from the outcome model was *b* = 0.115.

Following contemporary recommendations for mediation reporting, we did not classify the mediation pattern as partial or full based on the statistical significance of the direct association. Instead, we quantified the proportion of the model-implied total association represented by the indirect pathway. Using the direct association from the outcome model (*b* = 0.115), the professional-agency pathway represented 28.1% of the model-implied total association when AI self-efficacy was low, 39.5% at the mean level of AI self-efficacy, and 48.0% when AI self-efficacy was high. This pattern indicates that the relative contribution of the professional-agency pathway increased as AI self-efficacy increased, although the estimates should be interpreted as conditional associations rather than causal effects.

To formally test moderated mediation, we examined the index of moderated mediation. The index was positive and its bootstrap confidence interval did not include zero [Index = 0.045, Boot 95% CI (0.009, 0.089)], indicating that the indirect association between AI integration and teacher identity through professional agency became stronger as AI self-efficacy increased. Together, these findings support the hypothesized moderated mediation pattern.

## 5 Discussion

### 5.1 Overview of the main findings

Guided by the conceptual framework in Figure 1, this study examined how teachers' AI integration in instruction is associated with language teacher identity, and whether professional agency represents a theoretically specified statistical pathway in this association. The results converged on four core patterns. First, teachers who reported more frequent and higher-quality AI integration also reported a stronger sense of language teacher identity. Second, AI integration was associated with higher professional agency, and agency in turn showed a robust positive association with teacher identity, consistent with the view that identity consolidates through psychologically meaningful engagement with practice (Beijaard et al., 2004; Akkerman and Meijer, 2011; Varghese et al., 2005). Third, the indirect association between AI integration and teacher identity through professional agency was statistically supported, aligning with contemporary approaches to indirect effects in psychological models (Preacher and Hayes, 2008). Finally, AI self-efficacy was a significant moderator of the AI integration–professional agency association, yielding a first-stage moderated mediation pattern that is consistent with moderated mediation logic (Hayes, 2015).

Overall, these findings suggest that AI integration is not only a pedagogical choice but also a psychologically meaningful form of role enactment. When teachers reported that they could competently and intentionally mobilize AI, higher AI integration was more strongly associated with agentic functioning, and that agentic functioning was closely related to how teachers experienced themselves as language professionals.

### 5.2 AI integration as identity-relevant role enactment

Teacher identity is widely conceptualized as a dynamic sense of self that is continuously negotiated across professional expectations, personal values, and lived practice (Beijaard et al., 2004; Akkerman and Meijer, 2011). For language teachers in particular, identity work often involves ongoing interpretation of what it means to teach language well, how authority and expertise are performed, and how relationships with learners are shaped by classroom interaction (Varghese et al., 2005). Within this perspective, AI integration can be understood as an identity-relevant practice because it alters the conditions under which competence, expertise, and instructional judgment are experienced and displayed.

One psychologically plausible interpretation of the positive association observed here is that AI integration provides teachers with additional pathways to enact instructional competence and responsiveness, especially when AI tools are used to support formative feedback, diagnosis of learning needs, and personalization. Such enactments can function as identity-confirming experiences because they reinforce a sense of being effective, adaptive, and professionally legitimate. This interpretation is compatible with research emphasizing the role of teachers' beliefs and meaning making in shaping professional action and self-understanding (Biesta et al., 2015). It is also consistent with technology integration research showing that teachers' adoption is intertwined with confidence, beliefs about learning, and perceived cultural fit, all of which are psychologically proximal to identity processes (Ertmer and Ottenbreit-Leftwich, 2010).

At the same time, our model indicates that the association between AI integration and identity is not reducible to a single pathway. Even after accounting for professional agency, AI integration still showed a direct link with teacher identity. This pattern fits dialogical accounts of identity, where multiple voices and positions can be strengthened simultaneously through new practices, including positions such as innovator, designer of learning experiences, or reflective professional (Akkerman and Meijer, 2011). In this sense, AI integration may contribute to identity consolidation both through agency and through other psychological routes, such as perceived competence, professional recognition, or reduced friction in performing valued aspects of teaching.

### 5.3 Professional agency as a psychological mechanism linking AI integration to identity

The mediating role of professional agency is theoretically coherent when agency is viewed as a psychological capacity for intentional influence in one's work, including setting goals, reflecting on practice, seeking resources, and transforming routines (Eteläpelto et al., 2013). In educational work, professional agency has been framed as relational and context-sensitive, emerging from interactions between the person and the environment rather than being a fixed trait (Pietarinen et al., 2016). This relational framing helps clarify why AI integration, as a situated practice, can be linked to agency: AI use typically requires teachers to make consequential choices about when to rely on tools, how to evaluate outputs, and how to align tool-supported actions with pedagogical intentions. These choices invite, and sometimes demand, heightened intentionality and reflective regulation.

The professional-agency pathway represented a meaningful portion of the overall association. At the mean level of AI self-efficacy, the indirect pathway represented approximately 39.5% of the model-implied total association between AI integration and teacher identity, and this proportion increased from 28.1% at low AI self-efficacy to 48.0% at high AI self-efficacy. This suggests that professional agency was not merely a statistically detectable pathway, but a substantively relevant component of the observed association, particularly among teachers with stronger AI self-efficacy.

Our results therefore support a psychological story in which AI integration is associated with a stronger sense of agency, and agency is in turn strongly tied to identity. This is consistent with the idea that identity strengthens when individuals repeatedly experience themselves as authors of meaningful professional action, rather than passive implementers of external demands (Biesta et al., 2015; Eteläpelto et al., 2013). The magnitude of the agency-to-identity association observed in the outcome model also aligns with the broader view that identity consolidation is closely coupled with experiences of efficacy, influence, and coherence in professional roles (Beijaard et al., 2004).

This pattern is especially relevant for language teachers, whose work often hinges on micro-level interactional decisions and continuous calibration of instruction to learner responses (Varghese et al., 2005). In such contexts, AI integration may expand the repertoire of possible actions, but it also increases the need for teachers to actively curate, validate, and contextualize information. When teachers experience this expanded action space as something they can steer, agency becomes a psychologically central bridge between doing and being, linking practice to identity.

### 5.4 AI self-efficacy as a boundary condition for the AI integration–agency association

The moderation findings suggest that AI integration was more strongly associated with professional agency when teachers reported higher AI self-efficacy. This is consistent with social cognitive theory, which argues that efficacy beliefs shape whether individuals initiate actions, persist when difficulties arise, and interpret challenges as manageable rather than threatening (Bandura, 1977). In teacher psychology, efficacy has similarly been framed as a key belief system that organizes motivation and effort in instructional contexts (Tschannen-Moran and Woolfolk Hoy, 2001). Our findings are consistent with this logic in AI-mediated teaching by indicating that confidence in AI-supported teaching is not only related to AI exposure, but also marks a condition under which AI integration is more closely associated with agentic functioning.

The size of the moderation effect should also be interpreted carefully. The interaction term added a small amount of explained variance in professional agency beyond the main effects and covariates (Δ*R*^2^ = 0.008). This magnitude is consistent with the expectation that interaction effects in psychological survey data are often smaller than main effects. At the same time, the conditional indirect association increased from 0.045 to 0.106 on a 5-point teacher-identity scale, meaning that the indirect association under high AI self-efficacy was more than twice the corresponding association under low AI self-efficacy. Thus, the moderation pattern appears modest in absolute scale-score units but theoretically meaningful for understanding when AI integration is more closely linked to agency-related identity processes.

This moderation also fits self-determination theory perspectives emphasizing that perceived competence supports more self-endorsed engagement and adaptive regulation (Deci and Ryan, 2000). When teachers feel capable of using AI tools, AI integration may be experienced as an opportunity for self-directed improvement and experimentation. When teachers feel less capable, the same integration efforts may yield weaker gains in agency because cognitive load, uncertainty, or reliance on external cues can dilute the sense of personal authorship. The moderation pattern is also consistent with technology acceptance frameworks highlighting that beliefs about capability and usefulness shape adoption and sustained use (Venkatesh et al., 2003). Importantly, in the present psychological framing, the core point is not adoption as an endpoint but adoption as a context for self-regulation. AI self-efficacy appears to function as a psychological amplifier that helps teachers convert AI-related activity into a subjective sense of influence, initiative, and professional control.

Two domain-specific considerations make this amplification especially plausible in the current AI era. First, generative AI introduces epistemic uncertainty because outputs can be fluent yet incorrect, which makes critical evaluation central to competent use. Second, classroom use of AI can carry identity-relevant risks related to authenticity, expertise, and responsibility. These features likely raise the threshold for feeling agentic, thereby increasing the importance of efficacy beliefs. Recent discussions of large language models in education emphasize exactly these demands on educators, including the need for literacies, critical evaluation, and responsible integration (Kasneci et al., 2023; Kohnke et al., 2023). Under such conditions, AI self-efficacy becomes psychologically consequential because it shapes whether teachers experience AI use as supportive scaffolding for their own judgment or as a destabilizing force that undermines professional confidence.

### 5.5 Integrating the findings with contemporary work on AI in education

The study also contributes to emerging AI-in-education scholarship by offering a psychologically grounded account of why AI integration matters for teachers. Work in AI in education has called for stronger theory building that links technological change to learning and human functioning (Chen et al., 2020). The three-paradigm framing of AI in education further underscores that AI can function as a tool, a collaborator, or a system-level actor, each with distinct implications for agency and responsibility (Ouyang and Jiao, 2021). Our model is consistent with these perspectives in that it treats AI integration not as a monolithic behavior but as a psychologically meaningful practice that can reshape self-related constructs.

Notably, the mediator in this study is professional agency rather than an exclusively pedagogical variable. This helps align the manuscript with psychological science by foregrounding motivational and self-regulatory mechanisms through which AI-related behavior connects to identity. In addition, our findings resonate with recent self-determination theory accounts of how school support relates to teachers' technology integration and competence development, suggesting that technology-related growth is partially a function of teachers' psychological need satisfaction and perceived competence (Chiu, 2022; Chiu et al., 2024).

### 5.6 Implications for psychologically informed teacher development

The findings suggest several psychologically oriented directions for supporting teachers in AI-rich environments. First, if AI integration is linked to identity partly through professional agency, then interventions that strengthen agency-related processes may have downstream benefits for identity. Agency can be cultivated by supporting reflective practice, collaborative sensemaking, and opportunities for teachers to redesign routines in ways that they experience as self-authored (Pietarinen et al., 2016; Toom et al., 2017; Heikonen et al., 2017).

Second, the moderation results imply that strengthening AI self-efficacy may be a high-leverage target. From a social cognitive standpoint, efficacy grows through mastery experiences, vicarious learning, and credible feedback (Bandura, 1977). Practically, this means professional development that allows teachers to repeatedly succeed with bounded, meaningful AI tasks may be more psychologically potent than exposure-focused training. The relevance of domain-specific efficacy is also supported by recent measurement work on AI-supported teaching self-efficacy (Chou et al., 2024). When efficacy is strengthened, teachers may be more likely to interpret AI integration as an extension of their professional capability, thereby facilitating agency and identity consolidation.

Third, because identity is dialogical and socially situated, schools and professional communities can influence whether AI integration is experienced as identity-affirming. Contexts that legitimate experimentation while emphasizing responsibility and judgment may help teachers integrate AI into a coherent professional self. In language education settings, where communicative authenticity and interpretive expertise are core identity resources, discussions of ethical and effective use of conversational AI can be framed as part of professional identity work rather than as an external compliance task (Kohnke et al., 2023; Kasneci et al., 2023).

### 5.7 Limitations and future research directions

Several limitations should be considered when interpreting the findings. The cross-sectional survey design does not allow causal or temporal conclusions about the ordering of AI integration, professional agency, AI self-efficacy, and language teacher identity. Although the hypothesized model was grounded in prior theory and tested as a conditional process model, the results should be interpreted as evidence of associations and conditional indirect associations rather than causal effects. Longitudinal, experimental, and experience-sampling designs are needed to examine whether changes in AI integration precede changes in professional agency and teacher identity, whether agency and identity also shape later AI integration, and how these relations unfold over time.

A second limitation concerns the measurement of AI integration. Although the adapted 8-item scale showed good internal consistency, factor structure, and item-total correlations in the present sample, it was originally derived from a broader technology integration measure. Therefore, it may not fully capture generative-AI-specific teaching practices, such as prompt design, evaluation of AI-generated outputs, management of epistemic uncertainty, and ethical decision-making in AI-supported instruction. Future studies should further develop and validate AI integration measures that explicitly reflect these emerging generative AI practices.

A third limitation concerns the set of control variables included in the analysis. Although we controlled for key demographic and professional background variables, including gender, years of teaching, highest degree, school location, and AI-related training, other potentially relevant individual and institutional factors were not measured. For example, teachers' general self-efficacy, digital competence, technology anxiety, perceived school technical support, leadership support, and school-level AI policy climate may also be associated with AI integration, professional agency, and teacher identity. Future research should include a broader set of psychological and school-contextual covariates to examine whether the observed associations remain robust when these additional factors are considered.

A fourth limitation concerns sampling and generalizability. Participants were recruited through online teacher communities and school-based English teaching groups rather than through probability sampling from a complete sampling frame. Therefore, the findings should be interpreted as applying primarily to in-service high school English teachers who were reachable through similar online or school-based teaching communities and who voluntarily completed an online questionnaire. The results should not be interpreted as nationally representative estimates of all high school English teachers. Future studies should use broader sampling frames, school-level stratification, or multi-region probability-based designs to examine the generalizability of the present findings.

The present model motivates several directions for future work that can deepen psychological understanding of teachers' adaptation to AI. Longitudinal designs can clarify how identity and agency co-develop as teachers move from exploratory AI use to more stable integration patterns, and whether efficacy beliefs show reciprocal growth as suggested by social cognitive perspectives. Experience-sampling approaches may capture within-person fluctuations in agency as teachers encounter AI successes and setbacks, providing a fine-grained view of self-regulatory dynamics.

Further, differentiating forms of AI integration may be theoretically fruitful. For example, AI used for feedback, planning, or learner analytics may place different demands on judgment and thus engage agency in distinct ways. Extending the model to include social and contextual affordances could also align with relational accounts of agency, where the professional community and perceived support shape whether agency is enacted (Pietarinen et al., 2016; Chiu, 2022). Meta-analytic synthesis of educators' AI adoption factors also suggests that psychological determinants operate alongside contextual constraints, pointing to the value of integrative models that bridge individual differences and organizational conditions (Ma and Lei, 2024).

Finally, future work can examine identity tensions and values conflicts that may accompany AI integration. Dialogical identity accounts suggest that new tools can activate competing professional positions, such as innovator vs. guardian of disciplinary authenticity (Akkerman and Meijer, 2011). Understanding when such tensions lead to growth vs. fragmentation would further strengthen the psychological contribution of AI-in-teaching research.

## 6 Conclusion

This study advances a psychologically grounded account of how AI integration in instruction relates to language teacher identity among in-service high school English teachers. The results indicate that teachers who reported higher levels of AI integration also tended to report a more consolidated professional identity. Importantly, the indirect association through professional agency was statistically significant, suggesting that AI integration is linked to identity not only as a practical routine but also as an identity-relevant form of professional enactment associated with teachers' sense of being able to shape classroom work.

The findings further show that AI self-efficacy moderated the association between AI integration and professional agency, yielding a conditional indirect association with teacher identity. This pattern highlights the psychological significance of efficacy beliefs in the AI integration–agency association, while remaining consistent with the cross-sectional nature of the data. Overall, the model clarifies an associational pathway connecting practice, agency, and identity, and points to the value of supporting teachers' psychological resources alongside instructional competence in AI-mediated educational environments.

## Data Availability

The original contributions presented in the study are included in the article/supplementary material, further inquiries can be directed to the corresponding author.
